# Fabrication and Development of Binder-Free Mn–Fe–S Mixed Metal Sulfide Loaded Ni-Foam as Electrode for the Asymmetric Coin Cell Supercapacitor Device

**DOI:** 10.3390/nano12183193

**Published:** 2022-09-14

**Authors:** Jae Cheol Shin, Hee Kwon Yang, Jeong Seok Lee, Jong Hyuk Lee, Min Gyu Kang, Ein Kwon

**Affiliations:** Division of Electronics and Electrical Engineering, Dongguk University-Seoul, Seoul 04620, Korea

**Keywords:** mixed metal sulfides, Mn–Fe–S, fragmented nanofibrous structure, asymmetric coin cell device

## Abstract

Currently, the fast growth and advancement in technologies demands promising supercapacitors, which urgently require a distinctive electrode material with unique structures and excellent electrochemical properties. Herein, binder-free manganese iron sulfide (Mn–Fe–S) nanostructures were deposited directly onto Ni-foam through a facile one-step electrodeposition route in potentiodynamic mode. The deposition cycles were varied to investigate the effect of surface morphologies on Mn–Fe–S. The optimized deposition cycles result in a fragmented porous nanofibrous structure, which was confirmed using Field Emission Scanning Electron Microscopy (FE−SEM). X-ray photoelectron spectroscopy (XPS) confirmed the presence of Mn, Fe, and S elements. The energy dispersive X-ray spectroscopy and elemental mapping revealed a good distribution of Mn, Fe, and S elements across the Ni-foam. The electrochemical performance confirms a high areal capacitance of 795.7 mF cm^−2^ with a 24 μWh cm^−2^ energy density calculated at a 2 mA cm^−2^ current density for porous fragmented nanofiber Mn–Fe–S electrodes. The enhancement in capacitance is due to diffusive-controlled behavior dominating the capacitator, as shown by the charge–storage kinetics. Moreover, the assembled asymmetric coin cell device exhibited superior electrochemical performance with an acceptable cyclic performance of 78.7% for up to 95,000 consecutive cycles.

## 1. Introduction

As a new energy storage device, the ‘supercapacitor (SC)’ is one of the attractive energy storage devices considering its positive merits, such as ultra-fast charging/discharging, cyclic stability, high power density, and low weight [1]. SCs are used in a wide range of applications, e.g., hybrid electric vehicles, consumer electronics, and medical electronics. Based on the charge storage mechanism of electrode materials, SCs can be differentiated into the following two types: (1) the electric double-layer capacitor (EDLC) that accumulates charge at the interface of the electrode material and electrolytes—in short, carbon-based materials are used for EDLCs, which store charge by the physisorption of electrolytic ions, and do not undergo redox reactions; and (2) the pseudocapacitor that stores the charge by means of a reduction–oxidation reaction (Faradaic reactions) occurring at the electrode/electrolyte surface and sub-surface region [2,3]. Though EDLC devices are commercialized, they suffer from a low energy density and specific capacitance, which restrict their utilization for wider applications [4,5]. In contrast, pseudocapacitive materials, mainly metal-based materials (oxides, sulfides, carbides, and so on) and their composites have a high energy density with noticeable capacitance [6,7,8]. In the field of metal-based composites, transition metal sulfides (TMSs) have attracted considerable attention, owing to their rich redox-active sites, high electric conductivity (two orders higher than oxides), high theoretical capacitance, and superior cyclic stability, compared to metal oxides [9]. Considering the tremendous potential of TMSs, intensive work is being carried out on metal sulfides of TMS, Binary TMS, Mixed TMS, Layered TMS, Non-layered TMS, etc. [10,11,12]. Mixed TMSs show better electrochemical properties than the other TMSs due to their exclusive benefits, such as a rich structural chemistry, multi-electron redox reactions, low cost, and environment-friendliness [13,14]. Vivid synthesis techniques are reported for the preparation of mixed TMSs, such as hydrothermal [15,16], reflux [17,18], chemical bath deposition [19,20], and electrodeposition techniques [20,21,22]. Among all the synthesis techniques, electrodeposition is a simple method through which the controlled deposition of materials at room temperature (RT) is possible [23]. Moreover, using this method, the uniform deposition of electrode material can be carried out on conductive substrates of different shapes and size. The crystal structure, surface morphology, and thickness of the deposition material can be easily controlled by controlling the preparative parameters, such as precursor concentration, deposition time, deposition cycles, and applied potential window [24,25]. Various mixed TMSs, such as Ni–Co–S [26,27], Cu–Co–S [28,29,30], and Mn–Co–S [31,32], are widely studied, and have shown better electrochemical performance than the binary TMSs. Herein, we focused on Mn–Fe–S, considering the positive electrochemical benefits of both Mn and Fe, such as a high theoretical capacitance, good conductivity, and numerous mutually exclusive electrochemical active sites of Fe and Mn.

In this work, we introduce the Mn–Fe–S material for electrochemical storage application with a very simple one-step synthesis strategy using an electrodeposition route. It is reported and known that the surface morphology, deposition thickness, and respective electrochemical performance are influenced by the deposition cycles. For a few electrodeposition cycles, the deposition of material is lower, which decreases the electrochemical performance due to inadequate electrode material, while at a greater number of cycles, the electrochemical performance decreases, because of a decrement in the electrode material/electrolyte wettability. Thus, for full utilization of the electrode material, it is necessary to optimize the number of deposition cycles. Here, we systematically studied and optimized the deposition cycles in the potentiodynamic mode of Mn–Fe–S on nickel foam. The physicochemical and electrochemical properties of Mn–Fe–S with variations in electrodeposition cycles are studied in detail, and are included in the subsequent Sections.

## 2. Experimental Methods

Commercially available manganese(II) sulfate monohydrate (MnSO_4_·H_2_O), Iron(II) sulfate heptahydrate (FeSO_4_·7H_2_O), potassium hydroxide (KOH), and thiourea (NH_2_CSNH_2_) were used as precursor metal salts and as a reducing agent, respectively. Ethanol and acetone were used to clean the Ni-foam, which was purchased from Duksan, Ansansi, South Korea. These chemicals were purchased from Merk Pvt. Ltd., Seoul, South Korea, and were used as received. The solutions were made in deionised (DI) water.

Prior to deposition, the Ni-foam was cleaned with ethanol, acetone, and DI water under sonication treatment in each solution for 10 min. The Mn–Fe–S was electrodeposited on a 1 cm × 1 cm Ni-foam substrate, and used as a current collector. The 0.1 M MnSO_4_·H_2_O and FeSO_4_·7H_2_O were dissolved in 50 mL DI water. Furthermore, the 1 M KOH (to make pH of solution pH = 7), and excess NH_2_CSNH_2_ were added dropwise, and used as a reducing agent. The deposition was carried out using the potentiodynamic mode within a three-electrode system and with Ni-foam as the working electrode, graphite as the counter electrode, and Ag/AgCl filled with 3 M KCl (saturated calomel electrode (SCE)) as the reference electrode. The deposition of Mn–Fe–S on cleaned Ni-foam was performed using potentiostat (Metrohm autolab 302N, Utrecht, Netherland) at an applied potential of (−1 to 1.2) V vs. SCE with a 10 mV s^−1^ scan rate for an initial 10 cycles. After deposition, the sample was rinsed with DI water, and kept in an oven for drying at 70 °C for 6 h; thereafter, the sample was denoted MFS–10. The above experimental procedure was repeated for the deposition of Mn–Fe–S for 15 and 20 cycles, denoted as MFS–15 and MFS–20, respectively. These deposited samples were used for a detailed study of their physiochemical and electrochemical properties.

## 3. Characterization Techniques

The structural and chemical surface states of the Mn–Fe–S sample were investigated using X-ray diffraction (XRD; PAN analytical, Cu–Kα radiation) and X-ray photoelectron spectroscopy (XPS; K-alpha, Thermo Scientific, Dartford, UK), respectively. Surface morphology, elemental mapping, and elemental composition (energy dispersive spectroscopy (EDS)) were investigated by performing field emission scanning electron microscopy (FESEM, S-4800 HITACHI, Ltd., Tokyo, Japan).

The electrochemical measurement was conducted in the three-electrode (for the Mn–Fe–S@Ni-foam electrode) and two-electrode (asymmetric device) system. In the three-electrode system, deposited Mn–Fe–S@Ni-foam was used as the working electrode, graphite as the counter electrode, and saturated calomel electrode (SCE) as the reference electrode. However, in the two-electrode system, Mn–Fe–S@Ni-foam and AC@Ni-foam were used as the positive and negative electrode, respectively. The cyclic voltammetry (CV), galvanostatic charge–discharge (GCD), and electrochemical impedance spectroscopy (EIS) techniques were used for systematic investigation of the electrochemical performance of the Mn–Fe–S electrode.

## 4. Results and Discussions

### 4.1. X-ray Diffraction (XRD)

The XRD profiles of all the samples were recorded to understand the structural information of the as-prepared samples. The recorded XRD profiles of the synthesized samples are included in Figure S1 of the Supplementary Materials. In this figure, the only peaks that are observed that correspond to material belong to Ni-foam. Thus, the XRD confirmed that the formed material is amorphous in nature.

### 4.2. X-ray Photoelectron Spectroscopy (XPS)

**Figure 1:**
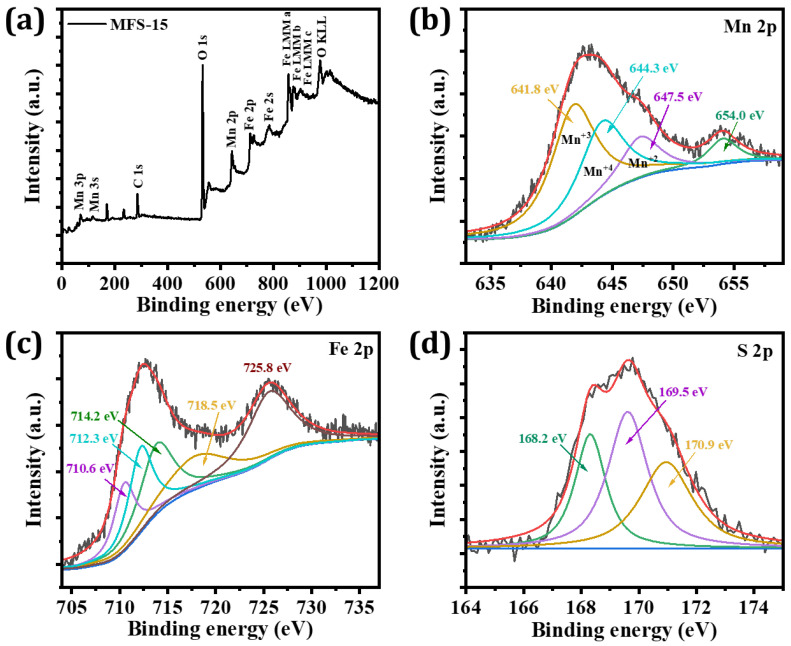
X-ray photoelectron spectroscopy of MFS-15 sample, (**a**) survey scan, (**b**) core-level spectra of Mn 2p, (**c**) core-level spectra of Fe 2p, and (**d**) core-level spectra of S 2p.

The compositional analysis of the sample was examined using the XPS technique. Figure 1a shows the survey scan spectrum of the optimized sample (MFS–15) recorded with a scanning range of (0–1200) eV. The scanned survey spectrum demonstrates the presence of Mn, Fe, O, and C, representing that only the elements of interest are present in the prepared sample. Furthermore, Figure 1b–d shows the core–level spectrum of each element that was deconvoluted to study the oxidation states of the present elements. Figure 1b shows the deconvoluted spectrum of the Mn 2p peak. This spectrum shows that the Mn 2p splits into doublets at 643.1 and 654.2 eV, corresponding to Mn 2p^3/2^ and Mn 2p^1/2^, respectively. The Mn 2p^3/2^ peak deconvoluted into three distinct peaks at 641.8, 644.3, and 647.5 eV, corresponding to Mn(III), Mn(IV), and Mn(II) states, respectively [30,31,33]. Figure 1c shows the Fe 2p narrow scan spectrum, which splits into two peaks at 712.5 and 725.8 eV that belong to Fe 2p^1/2^ and Fe 2p^3/2^, respectively. The peak corresponding to Fe 2p^3/2^ is enveloped by three peaks at binding energies of 710.6, 712.3, and 714.2 eV, indicating the presence of an Fe–O bond. Moreover, the peak at 718.5 eV is attributed to the satellite peak of Fe [34]. The deconvoluted spectrum of the S 2p core level is presented in Figure 1d. In this figure, the S 2p Gaussian peak splits into three peaks located at (168.02, 169.5, and 170.9) eV, corresponding to S^+6^, sulfate complexes, and bisulfate HSO_4_ complexes, respectively [35].

### 4.3. Field Emission Scanning Electron Microscopy (FE−SEM)

**Figure 2:**
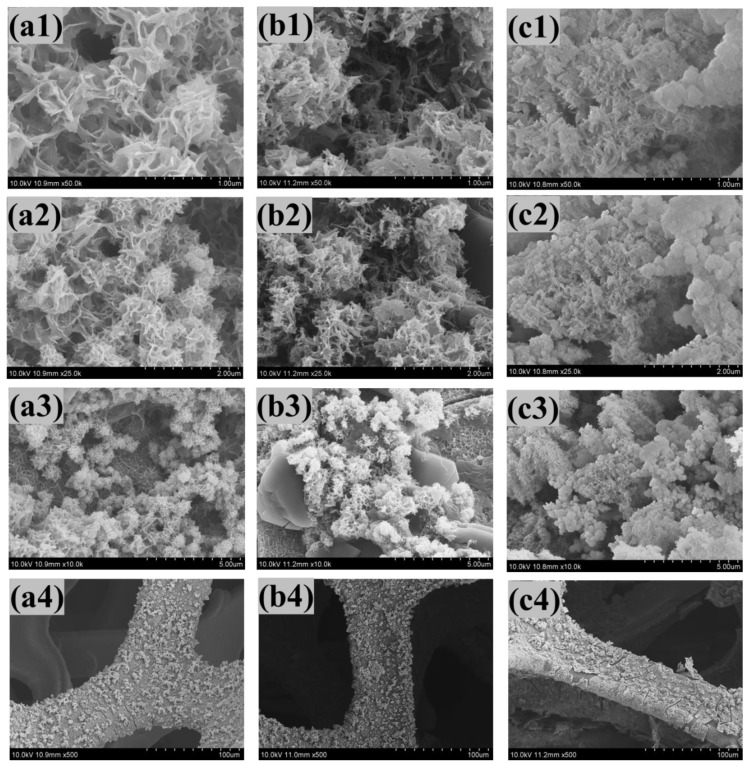
Field emission scanning electron microscope of (**a1**–**a4**) MFS-10, (**b1**–**b4**) MFS-15, and (**c1**–**c4**) MFS-20 sample at high to low magnifications (50kx;, 25kx, 10kx, and 5kx).

The morphological details of the synthesized Mn–Fe–S samples were obtained using FE−SEM imagery (as shown in Figure 2a–c). As can be seen upon close observation of the FE−SEM images of all samples, for the MFS–10 sample, a spongy mushroom type morphology initially formed (Figure 2(a1–a4)). In addition, as the number of potentiodynamic cycles increased from 10 to 15 cycles, a conversion in morphology from spongy mushroom to highly porous fragmented nanofibrous structure occurred (Figure 2(b1–b4)). The 1-D porous nanofibrous structure formed is beneficial for supercapacitive applications, as it helps to increase the surface-to-volume ratio, and supports easy ion permeability. Following a further increment in the CV cycles, i.e., for 20 cycles, the morphology again changes from fragmented nanofibers into agglomerated nanoparticles (Figure 2(c1–c4)). This agglomeration of nanoparticles decreases the surface area, thereby decreasing the effective electroactive surface. As can be seen by the increase in deposition cycles, it is considered that thickness of the deposited material increases which causes the surface to become rough. Furthermore, the porosity of material decreases with an increase in deposition cycles. The surface morphology of the electrode material is affected in terms of its electrochemical properties, such as the capacitance and electrode response. Thus, upon carefully observing the FE−SEM micrographs of all samples, the MFS–15 morphology appears to be superior to the other samples.

### 4.4. Energy-Dispersive X-ray Spectroscopy (EDS)

**Figure 3:**
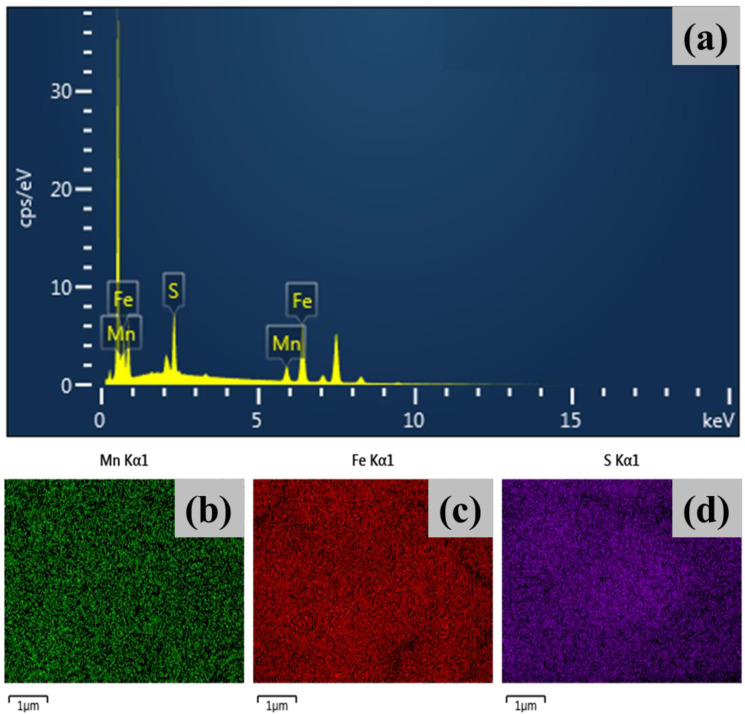
Elemental analysis of MFS-15 sample (**a**) energy dispersive spectroscopy (EDS) graph, elemental mapping analysis of (**b**) Mn Kα1, (**c**) Fe Kα1, and (**d**) S Kα1.

To study the compositional and distributional features of the material, the EDAX spectrum and elemental maps of all the Mn–Fe–S samples were recorded. Figure 3a shows the EDAX spectrum of MFS–15, while the EDAX of MFS–10 and MFS–20 are included in Figure S2a,b of the Supplementary Materials. In these spectra, the peaks corresponding to only the material of interest (Mn, Fe, and S) are seen. These results are consistent with the XPS analysis. To understand the distribution of each element, the elemental mapping results of the Mn–Fe–S samples were recorded. Elemental mapping images of MFS–15 are included in Figure 3b–d, and mapping images of the MFS–10 and MFS–20 samples are provided in Figure S2c–h of the Supplementary Materials. In all the elemental mapping images, Mn, Fe, and S elements are uniformly distributed across the surface of the substrate. This confirms that the uniform growth of Mn–Fe–S is synthesized on Ni-foam. Moreover, the atomic weight percentage ratio of Mn, Fe and S elements are included in Table S1 of the Supplementary Materials.

### 4.5. Electrochemical Measurements

The electrochemical properties of an electrode material are measured at RT in a three-electrode system using potentiostat Metrohm Nederland (autolab 302N). The measurements were carried out using cyclic voltammetry (CV), galvanostatic charge–discharge (GCD), and electrochemical impedance spectroscopy (EIS) techniques in 1 M aqueous KOH electrolyte. Additionally, for demonstration of its practical application, the coin cell asymmetric device was assembled using activated carbon as the negative electrode, and Mn–Fe as the positive electrode, which were separated by a polytetrafluoroethylene polymer (PTFE) membrane.

**Figure 4:**
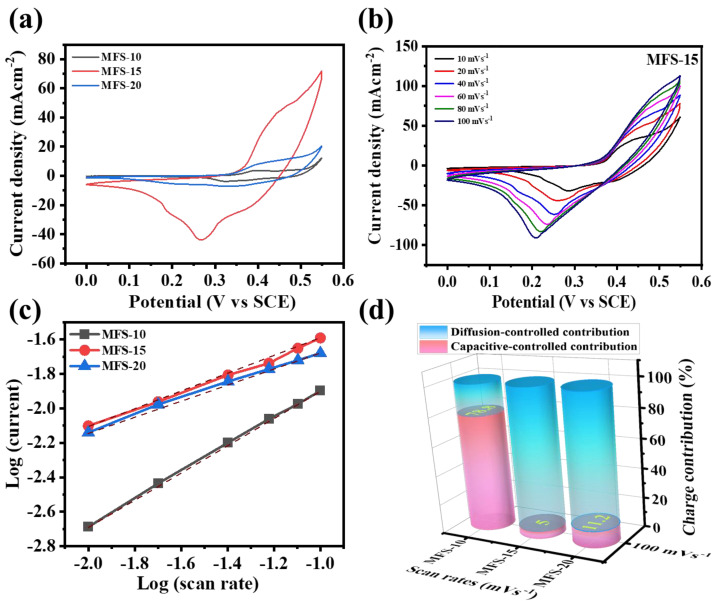
Cyclic voltammetry graphs measured in 1M KOH electrolyte (**a**) MFS electrode measured at 10 mV s^−1^ scan rate, (**b**) MFS-15 electrode at different scan rates (10–100 mV s^−1^), (**c**) Graph of log (current) vs. log (scan rate) for determining b–value, and (**d**) distinguishing capacitive and diffusive-controlled contribution of MFS electrodes at 100 mV s^−1^ scan rate.

CV was undertaken to determine the charge storage behavior of the electrode material. To determine the optimum deposition time of Mn–Fe–S electrodes (MFS–10, MFS–15, and MFS–15), the CVs were recorded at a 10 mV s^−1^ scan rate within a (0–0.55) V potential window. Figure 4a shows the redox peaks at both sides of the CV curves, which exhibit Faradaic charge storage behavior. The area under the CV curve of the MFS–15 electrode is greater than that of the other electrodes (MFS–10 and MFS–20). Detailed information about the optimized Mn–Fe–S electrode was obtained by measuring CVs at different scan rates of (10–100) mV s^−1^) with (0–0.55) V vs. SCE potential (Figure 4b). It was observed that the area under the CV curves increases with scan rates of (10→100) mV s^−1^, due to the fast electrode–electrolyte interface electrochemical kinetic reactions [36]. The shape of CV curves was not disturbed even at high scan rates, which demonstrates the reversibility of redox reactions [37].

To better understand the charge storage in Mn–Fe–S electrodes, the relation between the peak current and CV recorded at different scan rates of 10–100 mV s^−1^ were used (Figure S3a,b of the Supplementary Materials). Furthermore, the *b*-value can be calculated from the linear relationship between the current and applied scan rate (Figure 4c), using the following Equation (1) [38]:

(1) $$i=a\vartheta^{b}$$

(2) log(i)=log(a)+blog(ϑ)

where *a* and *b* are arbitrary constants, and *i* and *ϑ* are the peak current and scan rate, respectively.

In general, the value of ‘*b*’ predicts the charge storage of the electrode material, and whether it is diffusive- or capacitive-controlled. The *b*-value of 0.5 indicates diffusive behavior, while that of 1 represents the capacitive process [37,38]. For Mn–Fe–S, the b-value is (0.45 to 0.78), showing both diffusive and capacitive-controlled behavior for energy storage. The MFS–15 electrode (*b*-value 0.45) has a dominant diffusive-controlled process, as compared to other electrodes [39,40]. This means that the total current contributed during the charge storage process is a combination of the diffusion-limited process (intercalation of electrolytic ions inside electrode surfaces) and non-diffusive process (double-layer capacitance forming at the electrode–electrolyte interfaces) [41]. The exact quantitative distribution of the capacitive and diffusive-controlled contribution in percentage for the intercalation of K^+^ ions in Mn–Fe sulfide can be with by the Dunn method using Equation (3) [42,43] as follows:

(3) $$i=k_{1}\left(\vartheta \right)+k_{2}\left(\vartheta^{\frac{1}{2}}\right)$$

where *k*_1_*ϑ* and *k*_2_*ϑ*^1/2^ are the current contribution by means of the capacitive and diffusion-controlled processes related to ion intercalation within an electrode matrix. Here, *k*_1_ and *k*_2_ are the slope and intercept of the graph plotted using the *i/ϑ*^1/2^ vs. *ϑ*^1/2^ axes.

**Figure 5:**
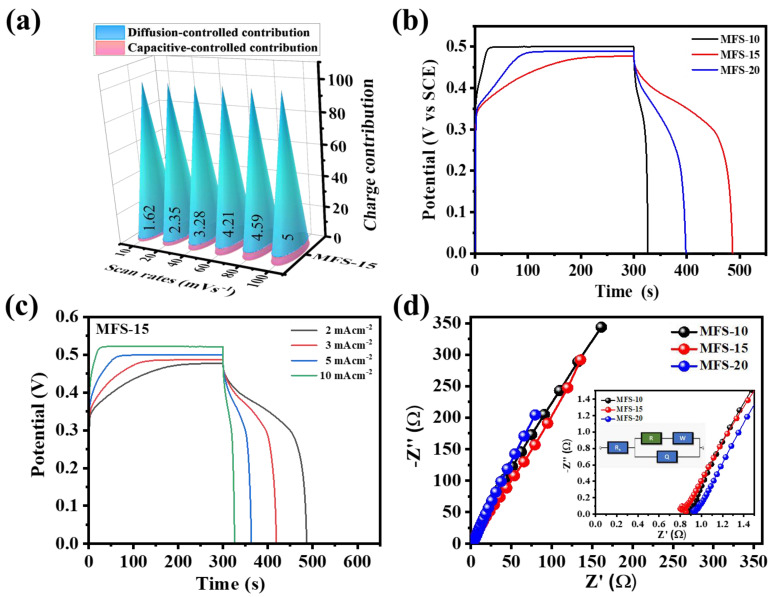
(**a**) Capacitive and diffusive -controlled contribution at different scan rates (10–100 mV s^−1^) for MFS-15 electrode, (**b**) galvanostatic charge–discharge (GCD) of MFS electrodes measured at 2 mA cm^−2^ current density, (**c**) GCD of MFS-15 electrode measured at different current densities (2–10 mA cm^−2^), and (**d**) electrochemical impedance spectroscopy of MFS-electrode measured at 10 mV potential within a 10^5^ to 0.1 Hz frequency range.

After defining the values of *k*_1_ and *k*_2_, the individual quantitative contribution of capacitive and diffusion-controlled processes can be calculated using Equation (2). The column graph of the diffusion- (aqua-blue region) and capacitive- (pink region) controlled contributions for Mn–Fe–S electrode material was calculated at a 100 mV s^−1^ scan rate, and is shown in Figure 4d. The diffusion-controlled contribution was observed to dominate the capacitive-controlled process for the MFS–15 and MFS–20 electrodes. For the MFS–10 electrode, the maximum charge stored by non-Faradaic (capacitive) behavior is due to the formation of flake-like nanostructures, which provide more surface sites to store charge [44]. The nanoflakes (MFS–10) were converted to a porous fragmented nanofiber (MFS–15)-like structure of Mn–Fe–S, which is beneficial for the intercalation of ions inside the 3D matrix of electrode material. Porous fragmented nanofibers contributed to the easy flow of electron for the appearance of a fast redox reaction at electrode surfaces. Figure 4d shows that the percentage of the diffusion-controlled contribution is 21.2, 95, and 88.8% for MFS–10, MFS–15, and MFS–20, respectively, measured at a 100 mV s^−1^ scan rate in 1 M KOH electrolytes. The capacitive-controlled contribution of the optimized MFS–15 electrode is observable at other scan rates of 10–100 mV s^−1^, as shown in Figure 5a. By considering the above results of charge storage kinetics, the electrochemical process in the Mn–Fe–S electrode is found to be a hybrid controlling process, in which the diffusion-controlled contribution is dominant, as compared to the capacitive.

GCD measurements were performed to calculate the quantitative parameters related to energy storage application. Figure 5b shows the GCD curves of Mn–Fe–S measured at a 2 mA cm^−2^ current density. During the GCD measurements, the charging time was kept constant (300 s), and due to this, the potential window for each electrode varied. This strategy was employed to avoid saturation of the electrodes, which depends on the applied current densities. The actual comparison of the energy storage electrode was based on input energy and output energy; therefore, during the measurement of GCD curves, the input charge (Q = i × t) was kept constant, and the discharge Q was recorded [37,45]. At low current density (2 mA cm^−2^), there is potential saturation for the MFS–10 electrode, whereas MFS–15 and MFS–20 show no considerable saturation potential. The areal capacitance (*C_A_*), energy density (*ED*), and power density (*PD*) of Mn–Fe–S were calculated using the following equations [3]:

(4) $$C_{A}\;\left({F\;\mathrm{cm}}^{-2}\right)=\frac{I_{d}\times T_{d}}{A\times dV}$$

(5) $$ED\;\left({\mathrm{Wh}\;\mathrm{kg}}^{-1}\right)=\frac{1}{2}C_{A}^{2}\times dV^{2}\times 3.6$$

(6) $$PD\;\left({W\;\mathrm{Kg}}^{-1}\right)=\frac{ED}{T_{d}}\times 3600$$

where *I_d_*, *T_d_*, *A*, and *dV* are the applied current (A) for the charging–discharging cycles, discharge time (s), deposited area of material (cm^2^) and kinetic potential (V) window of the electrode material, respectively.

The potential window, areal capacitance, and energy and power densities of the MFS–10, MFS–15, and MFS–20 electrodes were calculated using Equations (4)–(6), and are summarized in Table 1. The porous fragmented nanofiber structure has an areal capacitance of 795.7 mF cm^−2^ with a 24 μWh cm^−2^ energy density calculated at a 2 mA cm^−2^ applied current, which is considerably higher than that of the MFS–10 and MFS–20 electrodes. Figure 5c shows the GCD curves measured for MFS–15 sample at different current densities of 2, 3, 5, and 10 mA cm^−2^. Table 2 shows the calculated areal capacitance, energy density, and power density along with the applied potential window and current densities. The capacitance decreases at higher current densities because of the partial accessibility for electrolytic ions within the active material matrix. The MFS–15 electrode has ~60% retention in areal capacitance measured at high current densities from 2 to 10 mA cm^−2^.

An EIS study was employed to understand the charge transfer phenomenon at the electrode–electrolyte interface. EIS measurements of Mn–Fe–S electrodes were performed at a 10 mV bias potential within the 10^5^ to 10^−1^ Hz frequency range. Figure 5d shows Nyquist plots of the Mn–Fe–S electrodes used to derive the series resistance (R_s_) and charge transfer resistances (R_ct_) measured in a 1 M KOH electrolyte. The R_s_ is determined by the intercept of the real impedance (Z_re_) and diameter of the semicircle located at the high-mid-frequency region intercept to Z_re_, known as R_ct_ [46,47]. The values of R_s_ were determined by fitting the Nyquist plots using a Randle’s equivalent circuit, as shown in the inset of Figure 5d. Series resistance values of about 0.96, 0.85, & 0.93 Ω for MFS–10, MFS–15, and MFS–20 were determined, respectively. The detailed R_s_ values for Mn–Fe–S electrodes are shown in Table 1. A lower resistance is attributed to the higher wettability of the Mn–Fe–S electrode; additionally, the porous fragmented nanofibers ease the electron transfer path for ions [37].

Furthermore, the electrochemical performance of the Mn–Fe–S@Ni-foam electrode using the electrodeposition method was compared with other binary Mn-Fe oxide electrodes. Table 3 reports the synthesized materials, deposition method, developed nanostructures, specific capacitance, and respective stability of electrodes which were compared with this work (Mn–Fe–S electrode).

### 4.6. Asymmetric Coin Cell Device

Afterwards, the asymmetric coin cell (ASCC) device of Mn–Fe–S and activated carbon (AC) as a positive and negative electrode, respectively, was fabricated to evaluate the practical application of the electrode material. The negative (AC) electrode was prepared by mixing 80% activated carbon, 10% polyvinyl fluoride (PVDF), 10% carbon black, and 1-Methyl-2-pyrrolidinone (NMP) to make the slurry. This prepared slurry was pasted onto the cleaned Ni-foam, and dried at 80 °C for 4 h. Figure S4 of the Supplementary Materials shows the assembly of the coin cell device with all parts. The positive and negative electrodes were soaked by a 3 M KOH electrolyte, and separated by a Polytetrafluoroethylene (PTFE) membrane. The components of the device were all packed together in a coin cell 2032 under manual crimper at 1000 psi applied pressure. The balancing of charge (Q^+^ = Q^−^) was adopted using Equation (7) to calculate the mass ratio of electrode materials in the ASCC device [54]:

(7) $$\frac{m_{+}}{m_{-}}=\frac{C_{A}^{-}\times dV^{-}\;}{C_{A}^{+}\times dV^{+}}$$

where *m* is the mass of the electrode material, *C_A_* refers to areal capacitance, and *dV* refers to the potential window.

**Figure 6:**
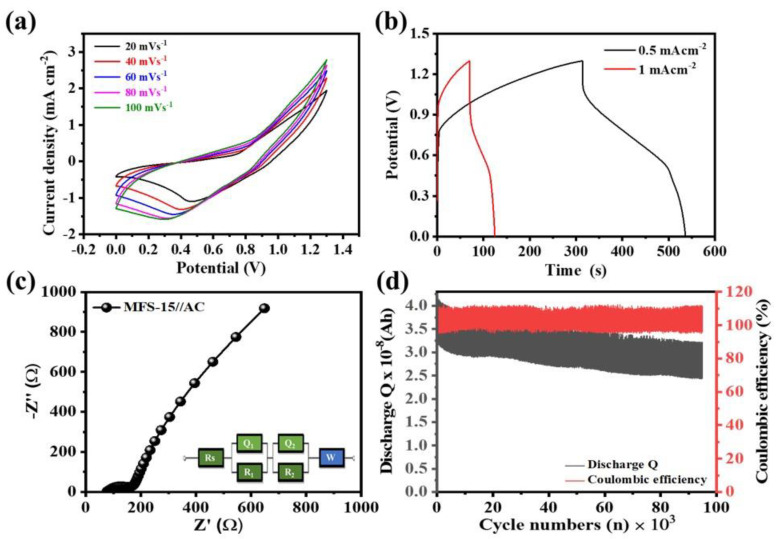
Asymmetric coin cell device of MFS-15 and AC electrode in 3M KOH electrolyte (**a**) CV at different scan rates (20–100 mV s^−1^), (**b**) GCD at different current densities (0.5–1 mA cm^−2^), (**c**) EIS at 10 mV potential within 10^5^–0.1 Hz frequency range, and (**d**) cyclic stability and coulombic efficiency measured up to 95,000 cycles based on discharge Q.

First, CV was carried out to determine the kinetic potential window of the ASCC device in a 3 M aqueous KOH electrolyte. The potential of the ASCC device can be a sum of the negative and positive electrodes in their respective electrolyte. Figure 6a shows the CV of the ASCC device measured at different scan rates of (20–100) mV s^−1^, which is operated within a 0–1.3 V potential. CV curves exhibit low intense redox peaks, and the area-under-the-curve increases at higher scan rates 20→100 mV s^−1^, without disturbing the shape, which is attributed to the pseudocapacitive nature. The GCD of the ASCC device was measured at different current densities of 0.5 and 1 mA cm^−2^, as shown in Figure 6b. The areal capacitance of the ASCC device is about 42.8 mF cm^−2^ with a 0.010 mWh cm^−2^ energy density at 0.5 mA cm^−2^ applied current density. Figure 6c shows the Nyquist plot of the ASCC device measured within a 10^5^ to 10^−1^ Hz frequency range at a 10 mV applied potential. The device shows resistance values of R_s_ and R_ct_ of about 76.4 and 99.7 Ω, respectively, while the inset of Figure 6c shows the best fitted circuit diagram of the ASCC device. The increase in the R_s_ value in the ASCC device is because of the contact resistances shown by the components of the coin cell device (solid–solid and solid–liquid interfaces). Importantly, the cyclic stability of the MFS–15//AC coin cell device was tested for continuous 95,000 GCD cycles at a 3 mA cm^−2^ current density. Figure 6d depicts the graph of the discharge Q (charge, Ah) and Coulombic efficiency (%) vs. cycle numbers (n) used to determine the retention in capacitance. In this study, the discharge Q of each cycle was calculated (up to 95,000 cycles), and the retention in capacitance and rate capability were calculated from the 1st to the 95,000th GCD discharge Q. It is observed that the capacitance decreases slowly with an increase in the GCD cycles. The ASCC device exhibits a cyclability of 78.7% along with a 105.3% Coulombic efficiency at up to 95,000 cycles. The decrease in capacitance may be due to a change in the microstructure and dissolution of metal ions in the liquid electrolyte [55,56].

## 5. Conclusions

The electrodeposition of Mn–Fe–S in the potentiodynamic mode was reported in this study. The amorphous phase of Mn–Fe–S was observed during RT deposition. A change in surface morphology was reported from spongy mushroom to fragmented fibrous-like nanostructures, due to the effect of deposition cycles. The Mn–Fe–S fragmented nanofiber-like structures provide low resistance, and provide easy pathways to the electron, which are beneficial for producing the best pseudocapacitive performance. We investigated the effect of the deposition cycles on electrode thickness and its direct consequence on the electrochemical performance. We noticed that for a lower number of deposition cycles, the charge storage ability is lower due to a lower amount of active material, while for a higher number of deposition cycles the charge storage capacity deteriorates due to reduction in the potential across electrode material. Due to reduction in the potential, the efficiency of the electrode material to bind ions in electrode pores decreases from the outer to the inner part, thereby reducing the capacitance. Moreover, for greater thicknesses of the electrode material the resistance of the material also increases, which hinders ion transportation. Thus, the optimized thickness of deposited material is necessary for achieving high energy storage capacity in electrodes.

## Figures and Tables

**Table 1:** Comparative table of areal capacitance, energy density, and power density and series resistance of Mn–Fe–S electrodes along with sample codes and applied potential window.

Sample Code	Potential Window (V)	Areal Capacitance (mF cm^−2^)	Energy Density (mWh cm^−2^)	Power Density (mW cm^−2^)	Series Resistance R_s_ (Ω)
**MFS-10**	0.50	108.0	0.0037	0.50	0.96
**MFS-15**	0.47	795.7	0.0244	0.47	0.85
**MFS-20**	0.49	400.0	0.0134	0.49	0.93

**Table 2:** Comparative table of areal capacitance, energy density, and power density of MFS-15 electrode calculated at different current densities along with potential window.

Current Density (mA cm^−2^)	Potential Window (V)	Areal Capacitance (mF cm^−2^)	Energy Density (mWh cm^−2^)	Power Density (mW cm^−2^)
**2**	0.47	795.7	0.0244	0.47
**3**	0.48	725.0	0.0232	0.72
**5**	0.50	630.1	0.0218	1.25
**10**	0.52	480.7	0.0181	2.6

**Table 3:** Comparison of previous reports on Mn-Fe binary metal oxides and sulfides as an electrode material for supercapacitor with present work.

Sr. No.	Material	Method	Nanostructure	Specific Capacitance	Stability	Ref.
**1**	Manganese-iron oxide	Electrospinning	Nanofibers	467 Fg^−1^ at 1 Ag^−1^	10,000 cycles—94%	[48]
**2**	Mn–Fe binary oxide	anodic deposition	Nano-spheres	280 Fg^−1^ at 5 mV s^−1^	-	[49]
**3**	MnFe_2_O_4_ RGO	Hydrothermal	Hollow sphere	768 F g^−1^ at 8 Ag^−1^	4000 cycles—95%	[50]
**4**	Mn–Fe-based alloys	electrodeposition	Nano-flakes	1.66 F cm^−2^ at 5 mV s^−1^	-	[51]
**5**	Mn−Ni−S	Electrodeposition	3D interconnected nanosheets	2849 Fg^−2^ at 1 Ag^−1^	11,000 cycles—90%	[52]
**6**	Fe–Mn–O nanocomposites	Chemical precipitation	Irregular spherical particles	125.2 Fg^−1^ at 0.2 Ag^−1^	1000 cycles—100%	[53]
**7**	**Mn–Fe–S**	**electrodeposition**	**fragmented porous nanofibrous**	**795.7 mF cm^−2^** **at 2 mA cm^−2^**	**95,000 cycles—78.7%**	**This work**

## Data Availability

The data presented in this study are available in [insert article or Supplementary Material here].

## Supplementary Materials

The following supporting information can be downloaded at: https://www.mdpi.com/article/10.3390/nano12183193/s1, Figure S1: X-ray diffraction pattern of MFS samples prepared at different deposition cycle; Figure S2: elemental analysis using energy dispersive spectroscopy of (a) MFS-10, (b) MFS-20, elemental mapping of MFS-10 and MFS-20 sample (c and f) Mn Kα1, (d and g) Fe Kα1, (e and h) S Kα1, respectively; Figure S3: Cyclic voltammetry measured in 1 M KOH electrolyte at different scan rates (a) MFS-10, (b) MFS-20, diffusion and capacitive charge contribution calculated at different scan rates (c) MFS-10 and (d) MFS-20 electrode; Figure S4: Schematic representation of coin cell assembly with their parts; Table S1: Data table for quantitative elemental distribution (wt. %) of MFS samples.
