# Supplementary material for: Fabrication and Development of Binder-Free Mn–Fe–S Mixed Metal Sulfide Loaded Ni-Foam as Electrode for the Asymmetric Coin Cell Supercapacitor Device
Source: Nanomaterials (Basel). 2022 Sep 14;12(18):3193. doi: 10.3390/nano12183193 (PMC9500630; doi:10.3390/nano12183193)
Supplement: Supplementary file 1 [file nanomaterials-12-03193-s001.zip › nanomaterials-1913461-supplementary.pdf]

Supplementary Materials

# Fabrication and Development of Binder-Free Mn–Fe–S Mixed Metal Sulfide Loaded Ni-Foam as Electrode for the Asymmetric Coin Cell Supercapacitor Device

**Jae Cheol Shin *, Hee Kwon Yang, Jeong Seok Lee, Jong Hyuk Lee, Min Gyu Kang and Ein Kwon**

Division of Electronics and Electrical Engineering, Dongguk University-Seoul, Seoul 04620, Korea
* Correspondence: author: jcshin@dgu.ac.kr

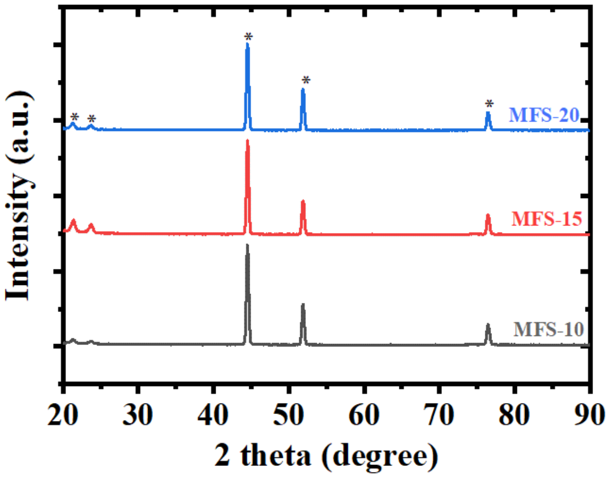


**Figure S1.** X-ray diffraction pattern of MFS samples prepared at different deposition cycle.

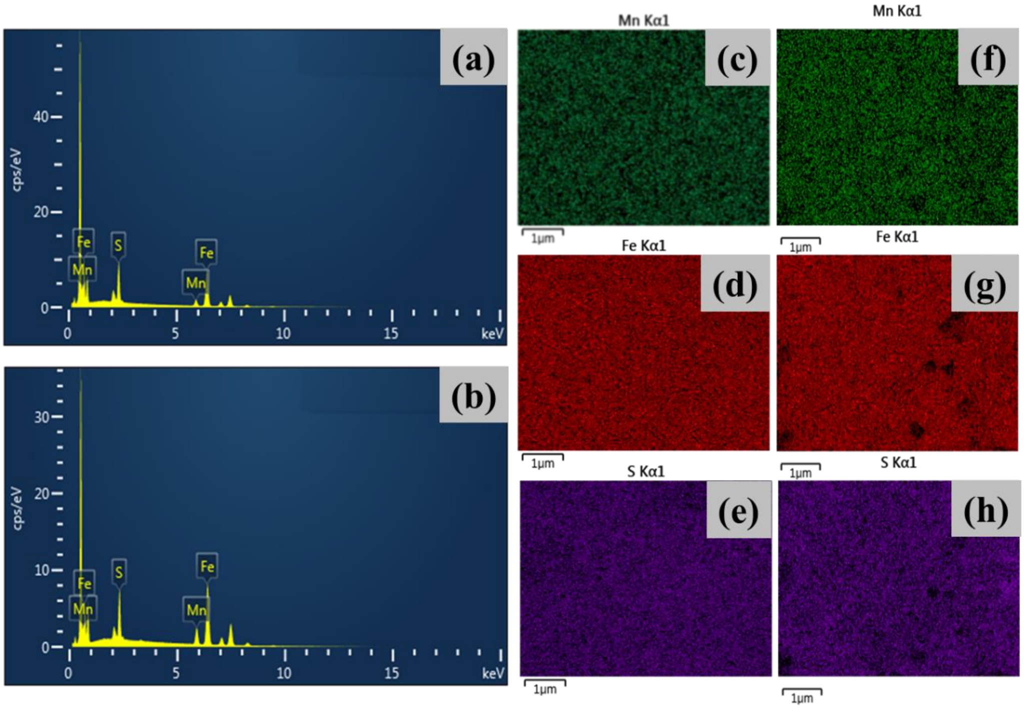


**Figure S2.** elemental analysis using energy dispersive spectroscopy of (a) MFS-10, (b) MFS-20, elemental mapping of MFS-10 and MFS-20 sample (c and f) Mn K$\alpha$1, (d and g) Fe K$\alpha$1, (e and h) S K$\alpha$1, respectively.

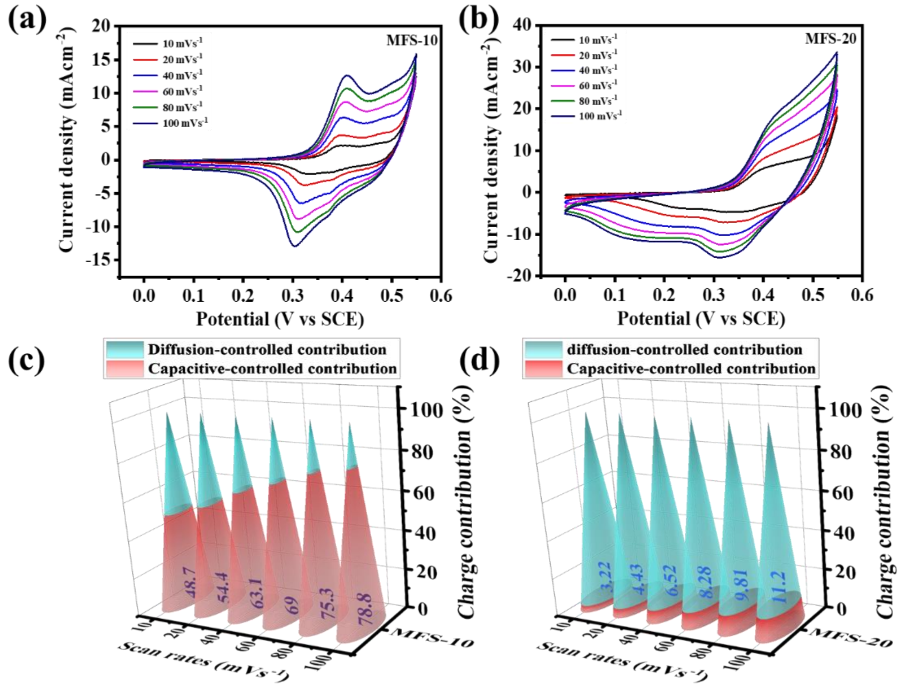


**Figure S3.** Cyclic voltammetry measured in 1 M KOH electrolyte at different scan rates (a) MFS-10, (b) MFS-20, diffusion and capacitive charge contribution calculated at different scan rates (c) MFS-10 and (d) MFS-20 electrode.

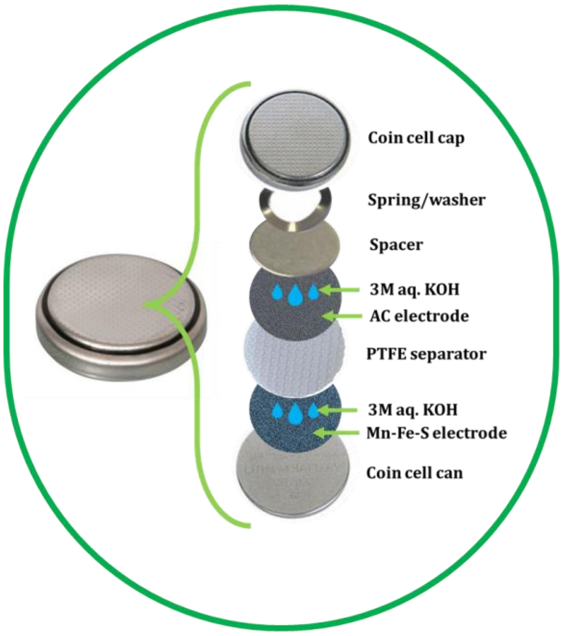


**Figure S4.** Schematic representation of coin cell assembly with their parts.

**Table S1.** Data table for quantitative elemental distribution (wt. %) of MFS samples.

| | Weight % | | |
|---|---|---|---|
| **Element** | **MFS - 10** | **MFS -15** | **MFS -20** |
| **S** | 14.76 | 13.65 | 10.75 |
| **Mn** | 10.91 | 15.29 | 16.71 |
| **Fe** | 74.34 | 71.06 | 72.54 |
| **Total** | 100.00 | 100.00 | 100.00 |
